# Energetic Cost and Kinematics of Pushing a Stroller on Flat and Uphill Terrain

**DOI:** 10.3389/fphys.2020.00574

**Published:** 2020-05-29

**Authors:** Øyvind Sandbakk, Rilana Perl, Hans-Christer Holmberg, Thomas Steiner

**Affiliations:** ^1^Centre for Elite Sports Research, Department of Neuromedicine and Movement Science, Norwegian University of Science and Technology, Trondheim, Norway; ^2^Section for Elite Sport, Swiss Federal Institute of Sport, Magglingen, Switzerland; ^3^Department of Health Sciences, Swedish Winter Sports Research Centre, Mid Sweden University, Östersund, Sweden; ^4^Department of Physiology and Pharmacology, Karolinska Institutet, Solna, Sweden

**Keywords:** endurance exercise, sex differences, running, walking, kinematics

## Abstract

During early parenthood, walking and/or running while pushing a stroller is a common form of endurance exercise among both recreationally active individuals and athletes. Here, we investigate how pushing a stroller influences the energetic cost, gross efficiency (GE), and kinematic behavior of well-trained men and women while walking or running on flat and uphill incline. Eight men and nine women, all recreationally active, performed three 5-min submaximal tests of walking or running during four different testing sessions, in randomized order: with and without pushing a 24.3-kg stroller on a flat (1%; 6, 8/9, and 11/12 km/h for women/men) and uphill (10%; 5, 6.5/7.5, and 7.5/8.5 km/h for women/men) incline. Respiratory parameters, heart rate (HR), blood lactate concentration, and rating of perceived exertion (RPE) were determined and video-based kinematic analysis was performed in connection with all these tests. Except while walking on the flat incline, pushing a stroller increased the energetic cost of walking/running under all conditions (all *p* < 0.05). This was associated with shorter and more rapid strides on both inclines (all *p* < 0.05); however, GE was higher when pushing the stroller (*p* < 0.05). The increase in energetic cost of pushing the stroller was approximately threefold higher uphill than on the flat incline, and women were influenced more than men when running uphill at the highest speed (all *p* < 0.05). Here, we provide novel insights on the energetic cost and kinematic behavior of pushing a stroller while walking or running on flat and uphill inclines. The energetic cost of pushing a stroller was clearly higher than for unloaded exercise, coincided by shorter and more rapid strides, and especially pronounced on uphill terrain where also women were more influenced than men.

## Introduction

**FIGURE 1:**
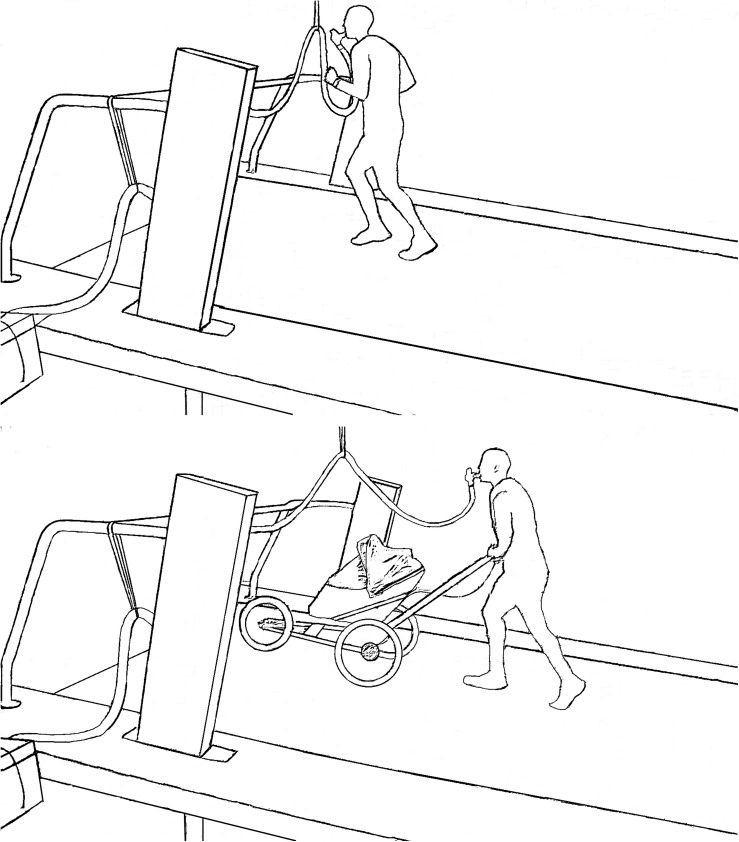
Comparison of stroller running and unloaded running while testing on the treadmill.

During early parenthood, when both recreationally active individuals and athletes have less time for physical exercise, walking and/or running with a stroller offers an alternative for maintaining or improving fitness (see Figure 1 for an illustration). However, research findings concerning physiological, kinematic, and perceptual responses to exercise with a stroller have been inconsistent (Smith et al., 2004; Gregory et al., 2012; Alcantara and Wall-Scheffler, 2017), making cost/benefit analysis of such exercise difficult. In addition, walking or running uphill while pushing a stroller, which is highly relevant to exercise in many areas, has not yet been examined.

Walking and running with a stroller at any given speed obviously involves more resistance than unloaded exercise. On flat terrain, this extra resistance is due mainly to rolling friction; whereas on uphill terrain more power is required to counteract gravity and larger fluctuations in kinetic energy occur. These different constraints leading to an increase in energetic cost and kinematic changes would be expected to increase with the incline.

Previously, the sub-maximal oxygen cost while pushing a stroller on flat terrain or running without a stroller was found to be similar (Alcantara and Wall-Scheffler, 2017). However, while running on more variable outdoor paths, with a constant requirement for additional power to handle continuous changes in incline, velocity, and direction, heart rate (HR) increased more with stroller running (Smith et al., 2004; Gregory et al., 2012). Furthermore, the weight of the stroller involved in previous studies varied considerably and the manner of pushing was not standardized.

Moreover, even though the energetic cost of stroller walking and/or running is expected to be greater than running with no load, it is unclear whether this difference is due solely to the stroller itself and/or alterations in efficiency of movement. In this context, gross efficiency (GE) provides a more valid measure of the efficiency of movement *per se*, and recently, Bolger et al. (2018) found that the GE decreased with additional distal weight during roller skiing on steep uphill, but not on relatively flat terrain. Similar findings have been reported concerning the weight of the rifle in biathlon (Stoggl et al., 2015). However, the impact of pushing a stroller on GE has yet to be investigated.

In this context, the movement pattern may influence GE, but there is currently no consensus concerning how stride length and rate are influenced by pushing a stroller. Although Alcantara and Wall-Scheffler (2017) showed a reduction in stride length while running at self-selected speeds, it was unknown whether changes in speed or pushing the stroller changed kinematic patterns. In contrast, Smith et al. (2004) observed no difference in either stride length or rate between stroller and unloaded running at similar speeds.

The average difference in the body mass of men and women is approximately 40% (Sandbakk et al., 2018), so that the relative mass of a stroller is greater for women. However, it has not yet been determined whether there is a sex difference in the influence of stroller walking or running on energetic cost and pattern of movement. The only study on this topic to date was published by Alcantara and Wall-Scheffler (2017), who found that male and female participants exhibited similar physiological and biomechanical changes when running with a stroller.

Accordingly, our major aim here was to determine how pushing a stroller while walking or running on flat or uphill terrain influences the energetic cost, GE, and kinematic behavior of well-trained men and women. We hypothesized that the energetic cost is greater, strides more rapid and GE unaltered when walking or running with a stroller in comparison to corresponding unloaded exercise. Moreover, we expected these effects to be more pronounced on uphill than flat terrain and for women compared to men.

## Materials and Methods

### Participants

A total of 17 recreationally active individuals (eight male and nine female) volunteered to participate in this study (Table 1).

**TABLE 1:** Characteristics of the participants (means ± SD).

	Men (*n* = 8)	Women (*n* = 9)
Age (years)	30 ± 4	30 ± 11
Body mass (kg)	78 ± 7	61 ± 8
Weekly exercise (h)	7 ± 4	7 ± 6
Weekly running (h)	3 ± 1	3 ± 3
Number of stroller walking/running sessions lasting >30 min	15 ± 35	23 ± 44
Number of weekly sessions of stroller running	10 ± 28	9 ± 27

### Ethics Statement

The Regional Committee for Medical and Health Research Ethics waives the requirement for ethical approval for this study. Therefore, the ethics of the study is done according to the institutional requirements and approval for data security and handling was obtained from the Norwegian Centre for Research Data. Prior to the data collection, all participants provided written informed consent to voluntarily take part in the study. The participants were informed that they could withdraw from the study at any point in time without providing a reason for doing so.

### Study Design and Procedures

Before each testing session, body height and mass were measured, followed by an individual 15-min familiarization with the treadmill and warm-up at low intensity. During each of four different sessions, each participant performed three successive 5-min submaximal walking and running tests unloaded or while pushing a 24.3-kg stroller on flat (1%) or uphill (10%) terrain, in randomized order (see Table 2). Two of these four trials were carried out on one day and the other two on a second day at least 48 h, but no longer than 8 days later. In connection with all tests respiratory parameters, HR, blood lactate concentration, and rating of perceived exertion (RPE) were determined and video-based kinematic analysis performed.

**TABLE 2:** The speeds and inclines at which our participants walked and ran with and without pushing a 24.3-kg stroller.

Sex	Mode of exercise	Incline	Walking (km/h)	Running (km/h)	Running (km/h)
Women	Without stroller	1%	6.0	8.0	11
Men			6.0	9.0	12
Women	With stroller	1%	6.0	8.0	11
Men			6.0	9.0	12
Women	Without stroller	10%	5.0	6.5	7.5
Men			5.0	7.5	8.5
Women	With stroller	10%	5.0	6.5	7.5
Men			5.0	7.5	8.5

The varying combinations of speed and incline employed here were based on extensive pilot testing of individuals whose state of fitness and training backgrounds were similar to those of our participants. Based on the findings of this pilot testing, the experimental protocol here was designed to achieve exercise of similar metabolic intensities in the men and women and to facilitate fast walking and running at gradually increasing speed on the two inclines. The 1% incline was chosen to simulate relatively flat terrain (instead of 0%), in order to compensate for the lack of air drag on the treadmill as suggested by Jones and Doust (1996). The 10% incline was the steepest that allowed both men and women to run naturally with the stroller at corresponding speeds.

The participants were given standard introductions, e.g., to hold the stroller with both hands and use only the gait specified (walking or running). The additional weight was 24.3 kg, i.e., 15.3 kg for the stroller plus 9.0 kg to simulate the weight of a toddler.

To ensure steady-state metabolic conditions, respiratory parameters were averaged over the last 2 min of every stage. Immediately after every stage, RPE was rated on the 6-20-point Borg scale, both as total effort (RPE_overall), and separately for the upper (RPE_arms) and lower extremities (RPE_legs). Thereafter, a blood sample was collected for determination of lactate concentration.

All tests were performed on a 5 × 3 m motor-driven treadmill (Bonte Technology, Zwolle, Netherlands), the speed and incline of which were calibrated regularly throughout the experiment with the six-camera Oqus motion capture system (Qualisys AB, Gothenburg, Sweden). The participants used a competition pro-series stroller (Emmaljunga Barnvagnsfabrik AB, Vittsjö, Sweden). Respiratory variables were measured using open-circuit respirometry. Expired gas was passed through a mixing chamber and analyzed continuously (Oxycon Pro, Jaeger GmbH, Hoechberg, Germany). The instruments were calibrated against ambient air and commercial gas containing known concentrations of O_2_ (16.00%) and CO_2_ (5.85%) before each test. The O_2_ and CO_2_ concentrations of ambient air were measured, and the flow transducer was calibrated using a 3-L high-precision syringe (Calibration syringe D, SensorMedics, Yorba Linda, CA, United States).

Heart rate was recorded with an HR monitor (Polar RS800, Polar Electro Oy, Kempele, Finland). Blood lactate concentration in 20 μL of blood taken from each participant’s fingertip was measured using the Biosen C-Line lactate analyzer (EKF Industrial Electronics, Magdeburg, Germany), calibrated once every 60 min with a 12 mmol⋅L^–1^ standard. Video-filming for kinematic analysis was performed with 60 Hz using a SONY Handycam HDR-CX625 (Sony Corporation, Tokyo, Japan) fixed beside the treadmill, to allow a full view of the participants and their entire range of movement. The error of the 2D spatial resolution employing the present setup was 19 mm according to the calculations presented by Padulo et al. (2014).

Work rate was calculated as the sum of power against gravity (P_g_ = m ⋅ g ⋅ sin α⋅ v) and friction (P_f_ = m ⋅ g ⋅ cos α ⋅ μ ⋅ v), where m is the combined weight of the participant (including stroller + “toddler”), g the gravitational acceleration, α the angle of incline, v the speed of the treadmill belt, and μ the frictional coefficient of the stroller (Sandbakk et al., 2010). The rolling friction force (F_f_) of the stroller was determined by using a towing test, and the friction coefficient (0.020) then calculated by dividing the friction force by the normal force (F_n_); μ = F_f_ ⋅ F_n_^–1^.

The metabolic rate was calculated as the product of *V̇*O_2_ and the oxygen energetic equivalent, using the associated respiratory exchange ratio and standard conversion tables (Peronnet and Massicotte, 1991). GE was calculated as the work rate divided by the metabolic rate and presented as a percentage for the 10% incline only (since we could not determine the work rate on the 1% incline accurately). Energetic cost (O_2_ mL⋅kg^–1^⋅km^–1^) was calculated as *V̇*O_2_ uptake (mL⋅kg^–1^⋅min^–1^) divided by the belt speed (km⋅h^–1^).

The stride rate (i.e., the number of steps performed per minute) was averaged over 18 strides while walking and 24 strides while running. One stride was defined as one foot touching the treadmill after the opposite foot had touched the treadmill. Stride length was determined by dividing the treadmill speed (m⋅s^–1^) by the stride rate.

### Statistical Analyses

All data were checked for normality using a Shapiro–Wilk test and are presented as means ± SD. A two-way repeated-measures ANOVA (speed × incline) was applied to evaluate potential global differences, as well as interaction effects; while a *post hoc* test with Bonferroni correction was applied to localize differences. For comparisons between conditions, the values without the stroller were always defined as 100%. Relationships between work rate and metabolic rate were analyzed by linear regression.

We compared men and women at equal speeds (i.e., 6 and 11 km h^–1^ on the 1% incline, and 5 and 7.5 km h^–1^ on the 10% incline). Since the men did not run at 11 km h^–1^, the comparisons at this speed were calculated using linear interpolation.

Statistical significance was set at an alpha level of <0.05. Statistical tests were processed using IBM SPSS statistics version 24 Software for Windows (SPSS Inc., Chicago, IL, United States) and Office Excel 2016 (Microsoft Corporation, Redmond, WA, United States).

## Results

While walking at the lowest speed on the 1% incline, pushing a stroller had no influence on physiological and perceptual variables; whereas when running on this same incline there was an interaction between conditions (i.e., stroller vs unloaded walking/running) and intensity (i.e., increase in speed) with respect to energetic cost and related cardiorespiratory parameters, HR, lactate concentration, RPE, and stride length/rate, all of which differed between conditions at the highest speed (Table 3; all *p* < 0.05). On the 10% incline, all of these parameters were altered significantly by the stroller at all intensities, except for RER and lactate concentration while walking at the slowest speed (Table 3; all other *p* < 0.05). In this case, no interaction effect of condition and intensity was observed. The relative difference between pushing a stroller and unloaded walking/running was approximately threefold greater on the 10% than 1% incline for all variables except stride rate at all intensities and RER and lactate concentration while walking (Table 3; all others *p* < 0.05).

**TABLE 3:** The physiological and kinematic behavior of men and women pooled while walking or running with and without a stroller.

		1% Incline						10% Incline					
		Walking (6.0 km/h)	*p*	Running (8/9 km/h)	*p*	Running (11/12 km/h)	*P*	Walking (5.0 km/h)	*p*	Running (6.5/7.5 km/h)	*p*	Running (7.5/8.5 km/h)	*p*
*V̇*O_2_ (mL⋅kg^–1^⋅min^–1^)	CON	17.5 ± 1.2	0.065	32.0 ± 2.1	<0.001	41.6 ± 3.2	<0.001	26.3 ± 1.4	<0.001	41.7 ± 2.8	<0.001	47.0 ± 3.5	<0.001
	STR	18.7 ± 1.8		34.6 ± 2.7		44.6 ± 4.0		32.4 ± 2.5		50.0 ± 3.9		55.6 ± 4.1	
RER	CON	0.89 ± 0.06	1.00	0.87 ± 0.04	1.00	0.94 ± 0.05	0.024	0.87 ± 0.04	1.00	0.92 ± 0.04	0.003	0.93 ± 0.06	<0.001
	STR	0.87 ± 0.06		0.89 ± 0.06		0.96 ± 0.06		0.86 ± 0.05		0.97 ± 0.06		1.00 ± 0.07	
Heart rate (bpm)	CON	103 ± 15	1.00	132 ± 17	0.010	157 ± 16	<0.001	119 ± 13	<0.001	150 ± 12	<0.001	163 ± 12	<0.001
	STR	106 ± 15		140 ± 17		164 ± 16		132 ± 12		168 ± 10		180 ± 10	
RPE_arms	CON	6 ± 0	1.00	8 ± 1	0.515	9 ± 2	0.545	7 ± 1	<0.001	8 ± 2	<0.001	10 ± 2	<0.001
	STR	7 ± 1		9 ± 2		10 ± 3		9 ± 2		12 ± 2		14 ± 2	
RPE_legs	CON	7 ± 1	1.00	10 ± 1	0.217	13 ± 2	0.054	8 ± 1	0.006	12 ± 2	0.011	14 ± 2	<0.001
	STR	8 ± 1		12 ± 2		14 ± 3		10 ± 2		14 ± 2		17 ± 1	
RPE_overall	CON	7 ± 1	0.228	9 ± 1	0.179	12 ± 2	0.248	8 ± 1	0.008	12 ± 2	0.016	14 ± 2	<0.001
	STR	8 ± 1		11 ± 2		13 ± 2		10 ± 2		14 ± 2		17 ± 1	
Lactate (mmol⋅L^–1^)	CON	1.02 ± 0.34	1.00	1.25 ± 0.49	0.341	2.60 ± 1.43	0.002	1.02 ± 0.32	1.00	2.01 ± 0.74	<0.001	2.91 ± 1.26	<0.001
	STR	1.05 ± 0.32		1.52 ± 0.71		3.55 ± 2.01		1.16 ± 0.30		3.86 ± 1.68		7.76 ± 3.36	
Stride rate (Hz)	CON	1.97 ± 0.1	0.095	2.68 ± 0.10	0.002	2.74 ± 0.11	<0.001	1.77 ± 0.10	0.040	2.63 ± 0.12	0.035	2.66 ± 0.11	0.001
	STR	2.00 ± 0.11		2.76 ± 0.09		2.86 ± 0.12		1.82 ± 0.13		2.68 ± 0.11		2.75 ± 0.10	
Stride length (m)	CON	0.85 ± 0.04	0.093	0.89 ± 0.07	0.003	1.17 ± 0.09	0.011	0.79 ± 0.04	0.046	0.75 ± 0.05	0.037	0.84 ± 0.06	0.002
	STR	0.84 ± 0.04		0.86 ± 0.06		1.10 ± 0.09		0.77 ± 0.05		0.73 ± 0.05		0.82 ± 0.06	
ECO (mL⋅kg^–1^⋅km^–1^)	CON	174 ± 11	0.062	226 ± 18	<0.001	216 ± 16	<0.001	315 ± 17	<0.001	354 ± 16	<0.001	350 ± 18	<0.001
	STR	188 ± 18		245 ± 20		233 ± 20		388 ± 30		425 ± 29		415 ± 26	
Gross efficiency (%)	CON							14.9 ± 0.7	<0.001	13.1 ± 0.5	<0.001	13.2 ± 0.6	<0.001
	STR							17.1 ± 0.8		15.3 ± 0.8		15.6 ± 0.7	

*All values presented are mean ± standard deviations. CON = control condition, walking/running without stroller; STR = stroller walking/running; V̇O_2_ = oxygen uptake; RER = respiratory exchange ratio; RPE = rating of perceived exertion; ECO = energetic cost.*

**FIGURE 2:**
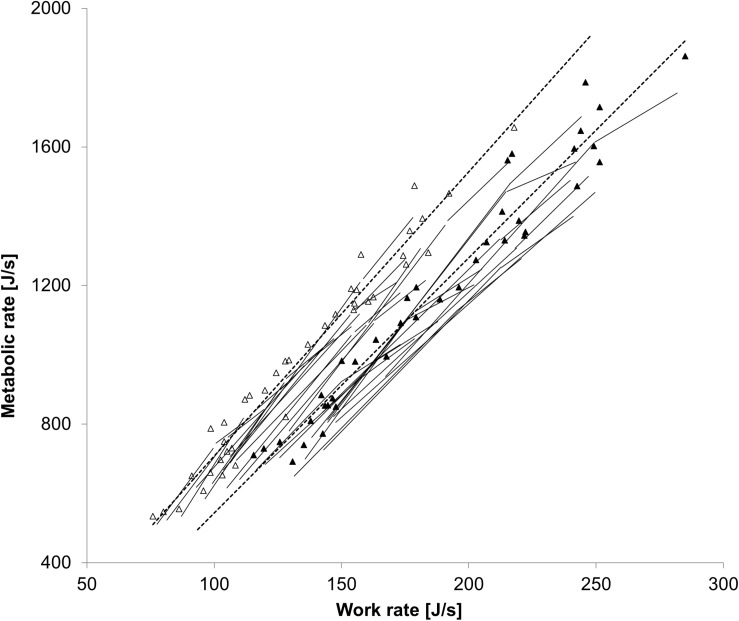
The relationship between the work rate and metabolic rate while walking/running with (▲) or without (Δ) a stroller on the 10% incline. Both individual values and average values of all participants (broken lines) are shown.

On the 10% incline, the metabolic rate at any given work rate was lower and the corresponding GE higher with stroller walking/running than with unloaded walking/running of all intensities (Figure 2; all *p* < 0.001).

The men took longer strides at lower rates than the women at both 6 and 11 km h^–1^ on the 1% incline, with lower RER, HR, and RPE_legs at 11 km h^–1^ (Table 4; all *p* < 0.05). On the 10% incline, RPE values were lower for the men while pushing a stroller at both speeds, whereas HR and lactate concentration were also lower in men at 7.5 km h^–1^ (Table 4; all *p* < 0.05). Furthermore, women demonstrated more pronounced increases in RPE, HR, and lactate concentration at 7.5 km h^–1^ on the 10% incline while stroller walking/running (*p* < 0.05).

**TABLE 4:** Comparison of the physiological and kinematic behavior of men and women while walking or running with or without a stroller.

		1% Incline						10% Incline					
		6.0 km/h			11 km/h			5.0 km/h			7.5 km/h		
		Men	Women	*p*	Men	Women	*p*	Men	Women	*p*	Men	Women	*p*
*V̇*O_2_ (mL⋅kg^–1^⋅min^–1^)	CON	17.2 ± 1.4	17.7 ± 1.0	0.325	39.0 ± 3.5	40.8 ± 2.1	0.262	26.3 ± 1.5	26.3 ± 1.3	0.702	43.6 ± 2.2	44.3 ± 1.2	0.969
	STR	18.1 ± 1.9	19.4 ± 1.5	0.123	42.4 ± 4.5	43.3 ± 1.6	0.655	31.1 ± 1.7	34.0 ± 2.5	0.089	51.8 ± 4.1	52.8 ± 2.5	0.966
RER	CON	0.89 ± 0.06	0.89 ± 0.06	0.870	0.90 ± 0.04	0.96 ± 0.04	0.001	0.86 ± 0.04	0.89 ± 0.03	0.228	0.92 ± 0.04	0.93 ± 0.05	0.527
	STR	0.87 ± 0.04	0.87 ± 0.08	0.971	0.92 ± 0.04	0.99 ± 0.05	0.006	0.86 ± 0.06	0.86 ± 0.03	0.927	0.97 ± 0.07	1.03 ± 0.07	0.365
Heart rate (bpm)	CON	97 ± 8	109 ± 17	0.088	140 ± 9	165 ± 18	0.002	117 ± 16	121 ± 9	0.877	151 ± 15	162 ± 9	0.518
	STR	102 ± 16	111 ± 13	0.195	149 ± 14	171 ± 16	0.002	128 ± 13	138 ± 9	0.411	167 ± 11	180 ± 11	0.243
RPE_arms	CON	6 ± 0	6 ± 1	0.351	8 ± 2	10 ± 3	0.031	6 ± 0	7 ± 1	0.102	8 ± 2	10 ± 2	0.176
	STR	7 ± 1	7 ± 1	0.285	9 ± 2	11 ± 3	0.108	9 ± 2	10 ± 2	0.158	11 ± 2	15 ± 1	0.020
RPE_legs	CON	7 ± 1	8 ± 1	0.279	11 ± 1	13 ± 1	0.005	8 ± 2	9 ± 1	0.158	12 ± 2	14 ± 1	0.121
	STR	8 ± 1	8 ± 1	0.451	12 ± 2	15 ± 2	0.043	9 ± 2	12 ± 1	0.030	13 ± 2	18 ± 1	0.003
RPE_overall	CON	7 ± 1	7 ± 1	0.351	11 ± 1	12 ± 2	0.080	8 ± 1	9 ± 1	0.224	12 ± 2	14 ± 1	0.421
	STR	8 ± 1	8 ± 1	0.763	12 ± 2	14 ± 2	0.243	9 ± 2	11 ± 1	0.017	13 ± 2	17 ± 1	0.004
Lactate (mmol⋅L^–1^)	CON	0.94 ± 0.27	1.10 ± 0.39	0.361	1.56 ± 0.54	3.43 ± 1.53	0.007	0.92 ± 0.30	1.16 ± 0.33	0.417	1.98 ± 0.87	2.89 ± 0.97	0.046
	STR	1.04 ± 0.27	1.06 ± 0.39	0.913	2.16 ± 0.95	4.51 ± 2.22	0.008	1.05 ± 0.34	1.30 ± 0.15	0.115	3.61 ± 1.62	9.42 ± 3.69	0.023
Stride rate (Hz)	CON	1.91 ± 0.06	2.03 ± 0.11	0.018	2.68 ± 0.08	2.80 ± 0.13	0.015	1.77 ± 0.03	1.77 ± 0.16	0.859	2.66 ± 0.11	2.64 ± 0.14	0.933
	STR	1.94 ± 0.05	2.07 ± 0.12	0.067	2.80 ± 0.09	2.91 ± 0.13	0.064	1.79 ± 0.07	1.82 ± 0.19	0.794	2.70 ± 0.08	2.77 ± 0.14	0.274
Stride length (m)	CON	0.87 ± 0.03	0.82 ± 0.05	0.018	1.14 ± 0.03	1.09 ± 0.05	0.014	0.79 ± 0.01	0.79 ± 0.07	0.988	0.78 ± 0.03	0.79 ± 0.04	0.886
	STR	0.86 ± 0.02	0.81 ± 0.04	0.060	1.07 ± 0.07	1.05 ± 0.05	0.640	0.78 ± 0.03	0.77 ± 0.08	0.905	0.77 ± 0.02	0.75 ± 0.03	0.301
ECO (mL⋅kg^–1^⋅km^–1^)	CON	173 ± 14	175 ± 8	0.626	213 ± 18	221 ± 11	0.344	315 ± 2	316 ± 15	0.793	349 ± 17	356 ± 10	0.695
	STR	181 ± 19	194 ± 16	0.118	232 ± 24	236 ± 10	0.690	374 ± 20	408 ± 31	0.095	415 ± 33	424 ± 22	1.00
Gross efficiency (%)	CON							14.9 ± 0.8	14.8 ± 0.7	0.868	13.3 ± 0.6	13.0 ± 0.4	0.727
	STR							17.3 ± 1.0	16.9 ± 0.7	0.556	15.3 ± 1.1	15.9 ± 0.3	0.089

*All values presented are mean ± standard deviations. CON = control condition, walking/running without stroller; STR = stroller walking/running; V̇O_2_ = oxygen uptake; RER = respiratory exchange ratio; RPE = rating of perceived exertion; ECO = energetic cost.*

## Discussion

Our present comparison of the energetic cost, GE, and kinematic behavior of well-trained men and women while walking and running with or without a stroller revealed that pushing a stroller increased the energetic cost under all conditions, except for walking on the flat terrain. The effects of pushing the stroller were greatest on the uphill terrain, where this additional load increased the energetic cost and related variables threefold more than on the flat. Pushing the stroller led to shorter and more rapid strides both on the flat and uphill incline; however, on uphill terrain the GE was higher with stroller exercise than without. Although most indicators of fatigue and effort were higher in the women than men, the influence of pushing a stroller on the women was more pronounced only at the highest speed on the steeper incline.

### Physiological and Perceptual Responses

Pushing a stroller increased the energetic cost of walking/running on both the flat and steep inclines, except for walking at the slowest speed on the 1% incline, where relatively little additional power is required to maintain speed, as also shown previously (Alcantara and Wall-Scheffler, 2017). In our case, pushing the stroller at higher speeds on the 1% incline enhanced the increase in energetic cost and related cardiorespiratory and perceptual parameters. This observation is in line with previous findings on running on more variable outdoor paths, where more power was required as the velocity increased (Smith et al., 2004; Gregory et al., 2012).

On the 10% incline, the increases in physiological and perceptual parameters while pushing the stroller were threefold greater than on flat terrain. While the added resistance on flat terrain is mainly due to more rolling friction, which is limited, pushing the 24.3-kg stroller uphill required more power against gravity, resulting in a greater increase in energetic cost and perceived effort.

The only exceptions noted were that RER and lactate concentration did not differ between unloaded and stroller exercise while walking at the slowest uphill speed. However, at such low intensity the participants were metabolically below the first lactate threshold, where neither lactate concentration nor indicators of utilization of fat vs carbohydrates as an energy source are expected to differ. Altogether, the cost of stroller walking/running was generally higher than with unloaded walking or running, an effect that was augmented on the uphill incline.

### Movement Efficiency

Although the extra cost of pushing a stroller was expected, whether this difference is due solely to the extra resistance caused by the stroller and/or movement efficiency is also altered has not been explored previously. Surprisingly, we found a higher GE during uphill walking/running with than without the stroller. Although the GE is influenced somewhat by the associated increase in work rate, due to the diminishing effect of the metabolic cost of zero work rate, this effect could not explain the more efficient movement pattern when pushing the stroller here. In fact, the GE was higher when walking at the slowest speed than when running at the two higher speeds.

One potential explanation for the greater efficiency with an extra load in the present study might be that participant’s pattern of movement when pushing the stroller involved less vertical displacement and more forward propulsion. Thus, although the ability to attain high speed when pushing a stroller is attenuated, the efficiency at submaximal speeds may be enhanced. In addition, pushing the stroller with both hands may involve less complex movements, thereby reducing energy cost.

### Kinematic Behavior

On both the flat and the uphill inclines, stroller walking and running involved shorter and more rapid strides at any given speed. Certain previous studies also showed this expected reduction in stride length, although these involved self-selected speeds, where it is unknown whether differences in speed itself and/or pushing the stroller alters kinematic patterns (Alcantara and Wall-Scheffler, 2017). However, in contrast to our data Smith et al. (2004) found no differences in stride length while walking unloaded or pushing a stroller at similar speeds. In the future, the influence of pushing a stroller on cycle parameters and more detailed biomechanical adaptions, as well as the effects of different pushing procedures require examination.

### Sex Differences

Although most indicators of fatigue and effort were generally higher in the women than men, the additional influence of pushing a stroller was only significantly more pronounced for the women at the highest speed on the uphill incline. This seems logical, as the relative effect of the added work rate against gravity when uphill stroller running is greater in women. However, in the only previous comparison of the effect of stroller walking/running on the physiology of men and women, Alcantara and Wall-Scheffler (2017) observed no significant differences.

## Conclusion

The present study provides novel insights into the influence of pushing a stroller on the energetic cost and kinematic behavior of walking and running on both flat and uphill inclines, an alternative form of exercise for both recreationally active individuals and athletes during early parenthood. As expected, the energetic cost of stroller walking and running was higher than that of unloaded exercise, coincided by shorter and more rapid strides, and especially pronounced on uphill terrain where also women were more influenced than men. Since GE was higher for stroller walking and running than without, the greater energetic costs with stroller walking and running was due solely to the added resistance of pushing the 24-kg stroller.

## Data Availability Statement

The datasets generated for this study are available on request to the corresponding author.

## Ethics Statement

The studies involving human participants were reviewed and approved by the Norwegian Centre for Research Data. The patients/participants provided their written informed consent to participate in this study.

## Author Contributions

All authors contributed in the design of the study. RP and ØS collected the data. RP, ØS, and TS performed the data handling and performed the statistical analyses. All authors contributed to the writing of the manuscript and to the design of figures and tables.

## Conflict of Interest

The authors declare that the research was conducted in the absence of any commercial or financial relationships that could be construed as a potential conflict of interest.
